# Structural Equation Models: From Paths to Networks (Westland 2019)

**DOI:** 10.1007/s11336-020-09719-0

**Published:** 2020-08-14

**Authors:** Marko Sarstedt, Christian M. Ringle

**Affiliations:** 1 Otto-von-Guericke-University Magdeburg, Universitätsplatz 2, 39106 Magdeburg, Germany; 2 School of Business and GA21, Monash University Malaysia, Subang Jaya, Selangor Malaysia; 3 Hamburg University of Technology, Hamburg, Germany; 4 University of Waikato, Hamilton, New Zealand

Structural equation modeling (SEM) is a statistical analytic framework that allows researchers to specify and test models with observed and latent (or unobservable) variables and their generally linear relationships. In the past decades, SEM has become a standard statistical analysis technique in behavioral, educational, psychological, and social science researchers’ repertoire.

From a technical perspective, SEM was developed as a mixture of two statistical fields—path analysis and data reduction. Path analysis is used to specify and examine directional relationships between observed variables, whereas data reduction is applied to uncover (unobserved) low-dimensional representations of observed variables, which are referred to as latent variables. Since two different data reduction techniques (i.e., factor analysis and principal component analysis) were available to the statistical community, SEM also evolved into two domains—factor-based and component-based (e.g., Jöreskog and Wold 1982). In factor-based SEM, in which the psychometric or psychological measurement tradition has strongly influenced, a (common) factor represents a latent variable under the assumption that each latent variable exists as an entity independent of observed variables, but also serves as the sole source of the associations between the observed variables. Conversely, in component-based SEM, which is more in line with traditional multivariate statistics, a weighted composite or a component of observed variables represents a latent variable under the assumption that the latter is an aggregation (or a direct consequence) of observed variables.

## About the Book

Westland’s (2019) *Structural Equation Models: From Paths to Networks* offers a concise, well-written, and non-technical reference for SEM. The textbook comprises 149 pages structured into eight chapters. The chapters are largely independent of one another, allowing them to be easily covered in a different order. The content of Westland’s (2019) textbook makes it very attractive as additional reading for methodological courses devoted to factor-based SEM and wanting to delve into the method’s historic roots. Readers will also appreciate the various overview tables offering excellent summaries of selected contents, as well as the book’s index, which allows them to quickly identify topics and key terms of interest.

Whereas most textbooks focus on either factor-based or component-based SEM (e.g., Hair et al. 2017; Kline 2016), Westland’s (2019) book is unique in that it showcases the full range of SEM methodologies, starting with Wright’s (1921) path analysis, followed by partial least squares (PLS) path modeling (e.g., the difference between it and PLS regression is explicitly addressed), full-information covariance-based SEM, and recent neural network-based approaches. By placing these methods in a historical context, the book provides insights into their similarities and differences (e.g., the Chicago School and the Scandinavian School), which certainly explain why some disciplines have clustered around one or the other SEM method.

This book has many other valuable aspects. Most notably, Westland (2019) strongly emphasizes research design issues, which often receive scant attention in textbooks. These issues have a fundamental bearing on the implications drawn from any SEM analysis (Rigdon et al. 2020). For example, Chapters 4 and 5 offer a detailed account of data adequacy in SEM, drawing specific attention to Likert scale data. Applied researchers will particularly benefit from the various guidelines for data collection and preparation, which go beyond the standard power analyses covered in popular textbooks. For example, Westland (2019, p. 101) derives a metric for computing sample sizes required to offset information loss through the use of Likert scales rather than continuous metrics. The explications in Chapter 7 on research protocols are particularly noteworthy. Common topics, such as the nature of models, theory building, and hypothesis testing, are presented concisely to offer scope for subjects one would not expect in a compact reference guide. For example, Westland (2019) issues cautionary warnings about behavioral biases, such as apriorisms, that impact causal inference. Given increasing concerns about reproducibility (Nuzzo 2015), users of SEM should take these warnings seriously, as causal inferential conclusions are only supported under specific conditions (Bollen and Pearl 2013).

## Future Improvements

Similar to any other textbook, there are also elements that we believe could be improved. While the structure is very clear, the chapters’ content distribution is sometimes uneven. For example, the author devotes an entire 22-page chapter (Chapter 2) and various sections (e.g., Chapter 4.4) throughout the 149-page book to PLS path modeling, only to conclude that “it is an ideal tool for unscrupulous or lazy researchers interested in bogus theories with random data” (Westland 2019, p. 38).

Apart from this structural concern, Westland’s (2019) conclusion underlines the general sentiment that component-based SEM and factor-based SEM are competing approaches. However, as suggested in other parts of the book, researchers’ functional background and adherence to a specific philosophy of science position contribute to the confusion over which method is “right” and which is “wrong” (Rigdon et al. 2017). These aspects have been extensively discussed in various social science fields (Henseler et al. 2014), including psychology (Rhemtulla et al. 2020; Rigdon et al. 2019). We believe a new edition would benefit from a more balanced description of the different viewpoints, given that recent advances in component-based SEM address the various concerns expressed in the book. For example, generalized structure component analysis offers a single optimization criterion as well as model fit metrics and allows researchers to impose model constraints (Hwang and Takane 2014). In addition, research has suggested extensions of component-based SEM methods that reliably estimate both factor- and component-model parameters (e.g., Dijkstra and Henseler 2015; Hwang et al. 2020).

Moreover, extensions of some of the simulations documented in the book would also be beneficial. For example, in Chapter 2, the author uses a simulation study to demonstrate the PLS path modeling method’s limitations. The simulation study assumes zero relationships between all the components, which is problematic, since the PLS path modeling algorithm—as also described in this textbook—requires a nomological net (i.e., at least some relationships that are not zero) to run adequately. The author’s general conclusion that “PLS path estimates are biased and highly dispersed from small samples” (Westland 2019, p. 36) should therefore only be considered in the context of this very specific model setup—see also Henseler et al. (2014).

## Conclusion

There is no doubt that a broad range of readers from varying disciplines will benefit from this book. By discussing often neglected topics in SEM, Westland (2019) offers the reverse of the various cookbook-type textbooks prevalent in so many social sciences fields. In light of this positioning, applied researchers with no or little knowledge of the topic need to be aware that they require an additional resource that offers practical guidance on how to apply SEM with programs such as LISREL or Mplus, and how to counter problems commonly encountered in SEM analyses. Despite the aspects that need improvement, *Structural Equation Models: From Paths to Networks* is a valuable resource for novice users who want to work with SEM. It also offers new perspectives for routine SEM users who wish to use this textbook to gain an overview of SEM’s underlying research philosophy, the broad portfolio of methods, their historical origins and interrelationships, recent developments, and other important topics such as data quality. Readers wanting to participate in the factor- vs. component-based methods debate should, however, look beyond the book and consider recent discussions on this topic (Petter 2018). Priced at about 170 USD for the hardcover version, the book is certainly in the upper price range, but most academic institutions will probably have access to the e-book version via SpringerLink.
